# “When I sleep under the net, nothing bothers me; I sleep well and I’m happy”: Senegal’s culture of net use and how inconveniences to net use do not translate to net abandonment

**DOI:** 10.1186/1475-2875-13-357

**Published:** 2014-09-12

**Authors:** Sara Berthe, Dana Loll, Sylvain L Faye, Issa Wone, Hannah Koenker, Bethany Arnold, Rachel Weber

**Affiliations:** Johns Hopkins Bloomberg School of Public Health Center for Communication Programs, Baltimore, MD USA; University of Michigan, Ann Arbor, MI USA; Department of Sociology, University Cheikh Anta DIOP, Dakar, Senegal; Department of Public Health, University Cheikh Anta DIOP, Dakar, Senegal; JHPIEGO, Baltimore, MD USA

**Keywords:** Malaria, Senegal, Bed net, LLIN, ITN, Qualitative, Barriers, Net culture

## Abstract

**Background:**

Despite recent advances in the fight against the disease, malaria remains a serious threat to the health and well-being of populations in endemic countries. The use of long-lasting insecticidal nets (LLIN) reduces contact between the vector and humans, thereby reducing transmission of the disease. LLINs have become an essential component of malaria control programmes worldwide.

**Methods:**

The Culture of Net Use study used qualitative and quantitative methods in a longitudinal and iterative design over two phases, in order to capture changes in net use over a year and a half period and covering both dry and rainy seasons. Data were collected from a total of 56 households in eight regions to understand variations due to geographical, cultural, and universal coverage differences. At the time of the data collection, the universal coverage campaign had been completed in six of the eight regions (Dakar and Thies excluded).

**Results:**

Perceived barriers to use were primarily related to the characteristics of the net itself, include shape, insecticide, and a variety of minority responses, such as perceived lack of mosquito density and being unaccustomed to using nets. Insecticide-related complaints found that insecticide did not present a significant barrier to use, but was cited as a nuisance. Feelings of suffocation continued to be the most commonly cited nuisance. Respondents who favoured the use of insecticide on nets appeared to be more aware of the health and malaria prevention benefits of the insecticide than those who perceived it negatively.

**Conclusion:**

Despite prior evidence that barriers such as heat, shape, insecticide and perceived mosquito density contribute to non-use of LLINs in other countries, this study has shown that these factors are considered more as nuisances and that they do not consistently prevent the use of nets among respondents in Senegal. Of those who cited inconveniences with their nets, few were moved to stop using a net. Respondents from this study overcame these barriers and continue to value the importance of nets.

## Background

Despite recent advances in the fight against the disease, malaria remains a serious threat to the health and well being of populations in endemic countries. In 2012, the World Health Organization (WHO) estimated a total of 207 million cases of malaria globally, which resulted in 627,000 deaths [1]. The use of long-lasting insecticidal nets (LLIN) reduces contact between the vector and humans thereby reducing transmission of the disease and LLINs have become an essential component of malaria control programmes worldwide [2]. Between 2004 and 2010, the number of insecticide-treated nets (ITN) distributed in sub-Saharan Africa increased rapidly from 6 million to 145 million nets and the percentage of households with at least one net rose to 54% [3].

Malaria is endemic in Senegal and is one of the main causes of morbidity and mortality in the country [1]. The highest percent of confirmed cases are in the southern and eastern parts of the country, though 11 of the northern districts are considered low-transmission with less than 5 cases per 1,000 population [4]. The 2010 Senegal Demographic and Health Survey found that 28.9% of the population used an ITN the previous night, while the percentage of the population with access to an ITN in their household was 32.1% [3]. The percentage of households owning at least one ITN was 66.2. The overall ratio of use to access, an indication of to what extent nets are used when they are available in the household, was 0.76. While a ratio of 0.76 indicates that a large majority of people with access to a net are using it, some nets do go unused. An unpublished post campaign survey conducted in Senegal in 2011 reported that in households that had one net per sleeping space, only 9.8% of people did not use a net the previous night; in these same households, 16.7% of nets went unused the previous night; the reasons given were mainly (55%) that the usual user was absent or the net wasn’t needed. Net non-usage was higher in urban than rural areas [5].

While there is no widely agreed upon definition of “net culture”, it has been defined in the past as an area or country where nets have “traditionally been used” and where “net use is an expected normal, and seen as protective or beneficial in some ways (and not necessarily for malaria prevention or even to avoid nuisance biting)” [6]. This study proposes the following definition of “culture of net use”: when a personal and communal tendency to sleep with a bed net consistently becomes a socially accepted norm and habitual behaviour.

### Background on net distributions in Senegal and context for universal coverage

LLIN use in Senegal is high among those with access to a net. Table 1 shows indicators related to the use and access of ITNs and provides a context for the net ownership and use in Senegal. Previous comparisons of household ownership and individual use may have been misleading, since factors contributing to the gap between them were unclear. These contributing factors could have been behaviour-related or access-related. However, using this new indicator to assess access takes into account whether or not households have an adequate number of ITNs and are then using them. This data shows that in Senegal, 76% of the population with access to a net is using a net [7].

**Table 1:** **Use** ***versus*** **access of ITNs in Senegal**

Survey year	% of population that used an ITN the previous night	% of population with access to an ITN within their own household	% of households owning ≥1 ITN	Ratio of use: access
MIS 2008	22.9%	34.9%	60.4%	0.66
DHS 2010	28.9%	38.1%	66.2%	0.76

In 2009, Senegal distributed approximately 2.2 million LLINs nationwide, targeting children 6-59 months [8]. Following this campaign, household ownership of at least one LLIN increased to 82.3%; however, data collected during the dry season showed that only 60% of all households had one or more nets hanging and only 41.4% of the population reported sleeping under a net of any kind the previous night [9]. Beginning in 2010, Senegal’s National Malaria Control Programme (NMCP) implemented a rolling universal coverage distribution of LLINs, to provide one net for each sleeping space in a household. By the end of 2013, 6,874,114 LLINs were distributed in all 14 regions of the country. An accompanying communication campaign stressed the need for every member of the family to sleep under a net every night, and throughout the entire year [10].

While mass campaign distributions have been successful in achieving high rates of ownership of nets in many countries including Senegal, this does not necessarily translate into consistent use of the nets [11–13]. Recent studies have found that the most common reported reasons for non-use of nets included discomfort of nets primarily due to heat and the perception of low mosquito density [14–18], outdoor sleeping [16], fears about insecticide used in treated nets [16, 19] and difficulty hanging a net [16]. Isolated studies have found that shape [13] and net cost or lack thereof [20] can affect use rates.

## Methods

The Culture of Net Use (CONU) study used qualitative and quantitative methods in a longitudinal and iterative design over two phases, in order to capture changes in net use over a year and a half period and covering both dry and rainy seasons. Data were collected from a total of 56 households in eight regions to understand variations due to geographical, cultural, and universal coverage differences. At the time of the data collection, the universal coverage campaign (UCC) had been completed in six of the eight regions (Dakar and Thies excluded). A team of sixteen researchers was trained on the study objectives, study design, and ethical treatment of human subjects.

### Procedures

Phase I data were collected during the cool, dry season in January 2012 in the regions of Dakar, Fatick, Louga, and Kolda. Phase II data were collected in August 2012 during the hot, rainy season, adding two compounds in each of the original regions and six compounds in the additional regions of Kedougou, Thies, Ziguinchor, and Saint Louis.

Compounds eligible for the study owned at least one net and included an adult over the age of 18, who was asked to provide consent and contact information to allow the study team to locate or contact them for subsequent research phases. Study team members then drew a map of the compound, noting all structures and habitual sleeping spaces, as well as those used during the previous night, and noted whether or not a net was associated with the sleeping space. Data collectors were trained to probe about alternative places where family members slept (e.g., outside). Each sleeping space was assigned a number, and a quantitative questionnaire was administered for each sleeping space. In-depth interviews (IDI) and focus group discussions (FGD) were conducted to explore the respondents’ perspectives on barriers and motivators of net use, net allocation within families, net care and repair, and other topics related to net use. Interviews and FGDs were conducted in local languages, including Wolof, Pulaar, and Serere to allow for full participation from the subjects.

***Phase I:*** During Phase I of data collection, IDIs were conducted with the head of the household or his/her representative. In addition, FGDs were held at each compound with up to 10 male and female adult members of that compound. A total of 24 IDIs and 20 family-based FGDs were completed in Phase I. Data were collected on a total of 255 sleeping spaces, 148 of which had nets associated with them. Some of the results from Phase I alluded to potential barriers to net use, which were further investigated in Phase II.

***Phase II:*** Findings from Phase I informed the design of Phase II data collection. IDIs were no longer conducted with the head of household in the existing compounds. Instead, to diversify the perspectives captured, IDIs were conducted with a different family member over the age of 18 using a newly developed interview guide. FGDs were conducted only in the new regions at the community level. Two FGDs, homogenous by sex, were conducted in each of the new regions. In previously enrolled compounds, data collectors updated the maps of sleeping spaces, noting any changes to the compound since the last phase. The Phase II FGD guide integrated questions from the Phase I guides and added new topics for discussion, based on the results from Phase I. A total of 56 IDIs and 8 community-based FGDs were completed during Phase II. Data were collected on a total of 556 sleeping spaces, 394 of which had nets associated with them.

During each phase of data collection, qualitative data were audio-recorded and then transcribed and translated verbatim from the local language into French in Microsoft Word. Transcripts were then entered into ATLAS.ti and coded by a team of independent coders using a codebook of themes of interest. Sleeping space questionnaire data were entered into Excel and converted into STATA for analysis.

### Ethical considerations

Once eligibility criteria were verified, the head of the household or other representative was asked to provide consent for the participation of the compound in the study. Other compound participants, including the IDI participant, were then provided with information on the study and asked for their consent to participate. All FGD participants were asked to provide consent to participate in the study. Approval for each phase of research was secured from the Johns Hopkins University’s Bloomberg School of Public Health’s Institutional Review Board in Baltimore, Maryland and from the *Comité National d’Ethique pour la Recherche en Santé* in Dakar, Senegal.

## Results

### General knowledge/perception of malaria

During Phase I, respondents were asked about their general knowledge of malaria, including its causes and any other information they could provide. In general, most respondents were knowledgeable about malaria, stating that mosquitoes transmitted the parasite and describing prevention methods, especially sleeping with an LLIN.

Conducted during the dry season, Phase I results showed relatively high rates of net use. Of the sleeping spaces with nets that were occupied during the previous night, 72.5% of the nets were actually used that night. Conducted during the rainy season, Phase II results similarly showed that of the sleeping spaces with nets that were occupied the previous night, 86.6% of the nets were actually used that night.

Among the 37 nets (27%) from Phase I and 53 nets (13.4%) from Phase II that were not hung when researchers visited the household, participants provided a multitude of reasons as to why nets were not hung. Participants asserted that they took nets down during the day and put them back up in the evening; that nets were cumbersome to leave hanging; that the shape of the net was incongruent with their living space; that the net was dirty and had to be washed; that there were no mosquitoes; that the owner was away travelling; and that the net did not fit with the decor.

These high proportions of nets associated with sleeping spaces, the proportion of access to use, and reported use of nets show the importance placed on LLIN use. In general, respondents from this study expressed that they valued using LLINs for malaria prevention, based on their protective qualities as well as their effectiveness in preventing mosquitoes from biting. They often stated that prevention was better than treatment.

### Perceived barriers to net use

Perceived barriers to use were primarily related to the characteristics of the net itself, include shape, insecticide, and variety of minority responses such as perceived lack of mosquito density and being unaccustomed to using nets.

Sleeping spaces and sleeping conditions throughout the study areas were varied. They included beds with frames and mattresses, mattresses on the floor, mats on the floor, and a wide range of the number of people per sleeping space. The sizes and shapes of mosquito nets that had been distributed did not always accommodate these different spaces. LLINs distributed in the UCC were standard, rectangular nets with the exception of Kolda, where conical nets were distributed.

During Phase I, respondents cited frustration with the size and shape of free nets received from the UCC. In general, the nets were perceived as too small for their sleeping spaces. Preferences in shape, size and color varied amongst regions. In Dakar, conical nets were seen as better for families since more people could sleep under them, while rectangular nets were perceived as better for individual use. In Kolda, nets were seen as part of the home’s decor and the rectangular net was felt to give more depth and shape to the room. However, very few respondents cited shape as a major barrier to their use of nets.

In several cases, a mismatch in shape preference led the owner to spend money to transform/modify their nets or to purchase a new net. When asked about the shape of her purchased mosquito net, one female respondent from Dakar stated that her net needed to be transformed to facilitate use:

*‘It was rectangular. You know, before we repainted the house, we had rectangular nets. After the painting, the walls were too hard for nails; therefore, we used the cover of a bucket to transform the net into a circular shape’. – Female, Dakar – SHS Guediawaye, Urban*

During Phase II, respondents discussed preferences for either a conical or rectangular net in terms of ease of use, citing reasons such as ease of hanging, the amount of space the net takes up in the room, and uncertainty about the quality of their non-preferred net. The size of one’s net did not lead to non-use, but was rather an inconvenience since either not all of the family members could fit under one net – leading to a need to acquire additional nets – or the net was too short to be properly tucked in. There was no data to show how respondents responded to the nets not covering all family members.

Transformation of nets was most common in Louga, the region that had most recently benefited from the universal coverage campaign. Respondents who preferred the circular shape of net for ease of hanging or aesthetic reasons found ways to adapt their nets by adding circular frames at the top of the net to mimic a conical net. Some visited a blacksmith to create a circular metal frame, while others purchased materials such as plastic tubing, thread, and a needle from the market. Innovative solutions included the use of general household goods such as circular fan covers and cardboard boxes. Other respondents took their nets to the local tailor to have the tops of the nets converted to conical shape and reinforced, and to add extra fabric to the bottom of the net, making it longer and more easily tucked in. In Louga, one respondent described the way she transformed her net and the benefits of doing so:

*‘My mosquito net is rectangular, but I took a circular piece of iron to transform it into a conical shape to make it easier to attach. Also, I put a mattress on the floor for my children and I can protect them also; if I left the net rectangular, it would only cover one sleeping space’. – Female, Louga – Ndame Ngott, Rural.*

### Other perceived barriers to use

#### Insecticide

Results from data collected during Phase I were dominated by issues around insecticide, specifically irritation or discomfort as a potential barrier to net use; either bed nets had too much insecticide or too little insecticide. Based on these results, questions were added to the Phase II discussion guides to probe further on these issues. While the insecticide-related complaints remained the same, Phase II data found that insecticide did not present a significant barrier to use, but was cited as a nuisance. Feelings of suffocation continued to be the most commonly cited nuisance.

#### Positive perceptions of insecticide

Respondents who perceived insecticide positively all stated that the insecticide protected them from mosquitoes and from malaria. The insecticide was seen as a way of chasing the mosquitoes away, and of killing both the mosquitoes and other insects such as fleas, cockroaches and flies. Respondents also stated that net use contributed to a better night’s sleep since the need to fan oneself or worry about these nuisances greatly decreased. One exchange between a man from Kolda and the data collector shows the benefits the man perceives from his LLIN:

*‘Facilitator (F): In your opinion, what’s good about the insecticide?*

*Respondent (R): The insecticide is effective. If a mosquito approaches the net during the night, in the morning I can see many dead mosquitoes due to the insecticide.*

*F: And what else? What pleases you about the insecticide treated net?*

*R: It pleases me because when I sleep under the net, nothing bothers me. I sleep well and I’m happy’. – Male, Kolda – Guiero Yoro Bocar, Rural*

While these respondents were aware that the insecticide could cause discomfort, they had a better understanding of the proper care methods when first receiving a net and the need to air it out, in the shade, for several days.

*‘There are disadvantages if the person who receives [the net] does not know how to use it; they are given a mosquito net and think they can hang it without doing anything beforehand. The seller must explain to people how to use the net and tell them to put in the open air for some hours until the product is released’. – Male, Dakar – Deni Malick Gueye, Rural*

The respondents who favoured the use of insecticide on nets appeared to be more aware of the health and malaria prevention benefits of the insecticide than those who perceived it negatively.

#### Negative perceptions of insecticide

Feelings of suffocation, breathing difficulties and skin problems represented the majority of the negative perceptions of insecticide. However, in addition to these concerns, respondents mentioned general health consequences and bad odor of the insecticide. Of the respondents who reported problems with insecticide during Phase I, many stated that they stopped using a net following one of these major irritations and transitioned to using a different prevention method, such as a fan or coils.

Respondents in general reported that they did not know exactly what the product (insecticide) was, but knew that it probably was not good for their health, despite it being an effective mode of killing mosquitoes. One female from Kedougou stated:

*‘There’s a lot of insecticide. Sometimes it’s too strong and difficult to sleep under the net for the first time. The insecticide prevents me from breathing very well and it’s very difficult to breathe when around this product’. – Female, Kedougou – Kedougou Commune, Urban*

Other respondents were particularly concerned about children, who have weaker immune systems than their adult counterparts. One male from Ziguinchor states:

*‘We are victims. The product on our nets is unknown; we don’t know where it comes from. They come and give us the nets and after that, it creates illnesses such as colds and headaches. With the nets that are sold, I can see on the package that it comes from a lab and it was tested. People took time to study the nets and test the product inside. Yes, you get your net and your hang it in your home, but then numerous illnesses begin. In my opinion, they should tell people what product they are putting on the nets’. – Male, Ziguinchor – Djiringho, Urban*

Despite these perceptions and worries, most respondents were still using their nets. One respondent from Saint Louis stated that despite the negative effects of the insecticide, he continued to use his net:

*‘R: Once, I slept under a treated net with a high dose of insecticide and my arm and leg came into contact with the net. The next day, where my arm and leg touched the net were very hot. That’s how I know the dosage was high. Plus, my wife says similar things.*

*F: What did you do when you saw that your arm was hot?*

*R: Nothing. I just knew that the net had a high dose of insecticide which caused it.*

*F: And the net…what do you do with the net?*

*R: Despite everything, I continue to use it’. – Male, Saint Louis – Fanaye, Rural*

To overcome nuisances associated with the insecticide, a small minority of respondents decided to forego using a net; a larger majority chose to wash their net multiple times with a powder detergent (Omo) and/or bleach to help reduce/remove the insecticide before using the net again. These nuisances were found nationwide; no trends were found on a regional basis.

### Other barriers to net use

While insecticide was the most commonly cited nuisance in this study, two other barriers were also cited: perceived mosquito density and being unaccustomed to using nets. While some respondents cited sleeping with their net all year round, others stated that on average, they use their nets between five and seven months of the year, during the months surrounding the rainy season. These respondents perceived a lower mosquito density in the non-rainy season or were worried that the dust would dirty their nets and, therefore, chose to store them until the following rainy season. One male respondent from Ziguinchor states,

*‘F: You said that it was during the rainy season when there are more mosquitoes that you sleep under nets. Are there other seasons which you will not sleep under a mosquito net?*

*R: There is a small number of the population that sleeps with their nets throughout the year, but they are few. It’s sufficient enough to sleep with nets during the rainy season and once it is finished, everyone is free to sleep without nets.*

*F: And why is that?*

*R: It is believed that mosquitoes are harmful, or that one senses them, only during the rainy season. In the dry season, as I said before, we don’t notice mosquitos, except near areas of stagnant water, like the latrines’. – Male, Ziguinchor – Coubalan, Rural*

Other respondents stated that they were unaccustomed to using nets; for a variety of reasons, including laziness, frugality and wanting to preserve their net as long as possible, these respondents had rarely or never slept under a net and were not currently using them. Additionally, some respondents stated that though a net hung above their bed, they did not routinely pull them down at night to cover themselves.

These reasons for non-use were cited in a small minority of the interviews, with perceived lack of mosquito density standing out as the most significant. Being unaccustomed to using a net was not cited as a barrier *per se*, but was rather stated as justification for non-use.

## Discussion

This study reveals that the majority of respondents’ preconceived barriers to net use are not actual barriers to use, but rather nuisances or inconveniences to the user: of those who cited inconveniences with their nets, few were moved to stop using a net. Respondents from this study overcame these barriers and continue to value the importance of nets. This indicates that a strong culture of net use exists in Senegal.

Overall, net owners valued and understood the importance of using their net; the majority of respondents had a net, used it frequently and took care of it. Despite the occasional inconvenience of using a net – a non-preferred shape, bothersome insecticide, and perceived low mosquito density – respondents continually adapted to bridge the gap between what they received for free and what they needed for their specific situation. Use of nets appears to be grounded in a strong knowledge and awareness of malaria and malaria prevention. Respondents knew that malaria was caused by mosquitoes and knew that nets were the best prevention method.

While initial findings in Phase 1 suggested that people were abandoning their nets due to problems with the insecticide, probing on this issue in Phase II revealed that insecticide was more of a nuisance than a barrier and that after encountering a negative effect of the insecticide (burning skin, trouble breathing) that people continued using their nets, typically after airing them out. Initial reactions to the LLIN, including a bad odor and fear of the insecticide were time-limited barriers; after airing out or washing the net, discomfort diminished and use continued. Knowledge amongst respondents varied widely about how and when to air out nets and the amount of insecticide on their nets; the results globally showed a disagreement over whether purchased nets or free nets had a stronger level of insecticide. Throughout this study, some respondents stated that they received nets with no packaging, via the universal coverage campaign. Not only did this lead to perceptions of lower quality, but instructions on the packaging regarding use or information regarding the insecticide were lost as a result. While the national communication strategy contained messaging about proper care and repair and washing methods, and presumably distribution agents communicated these messages, not all key points appear to have been received. The distribution sites were often very busy with a variety of activities and it is possible, even likely, that not every LLIN recipient was present or able to hear these messages. Without hearing these messages and properly airing out nets, respondents may have thought that the insecticide was more dangerous than it really is. Additional communication strategies are needed, both at the time of net distribution and throughout the year, to ensure that the population receives consistent guidelines on proper net use and care and repair instructions. Together, these communication strategies could greatly reduce the perceived and actual nuisances caused by the insecticide and further improve use.

While respondents expressed preferences for certain shapes to facilitate hanging or for aesthetic reasons, mass campaigns only provide one type of net. In Senegal, the general preference appeared to be for conical nets, though respondents from Kolda stated that they preferred rectangular nets. In order to transform their nets into their preferred shape, people were observed using a variety of locally available items. Some households added length and reinforced their nets by sewing additional fabric at the bottom, allowing for personalization of the nets with particular fabrics. This transformation activity has great implications for donors and programmes ordering nets for distribution activities. While the Global Fund and PMI have strict procurement guidelines for ordering nets, National Malaria Control Programmes may feel pressured to accommodate the preferences and interests of their populations. However, if a single type of net can be procured and families can easily transform and personalize these nets, this alleviates the pressure and potential financial burden on programmes to cater to different preferences.

Previous studies from around the globe show that a variety of barriers to net use exist. Often the use or non-use of LLINs is a multi-causal phenomenon and complex; the causes are not just from one variable, but more often at the crossroads of several factors. While the literature cites reasons such as heat, perceived mosquito density and fear of insecticide as barriers to use [14–18], the results of this research show that while these are valid nuisances to use among study respondents, they do not usually cause users to abandon use of nets in the Senegalese context. Instead, users are adapting to these nuisances and continuing to use LLINs for malaria prevention.

The heat associated with using a mosquito net has often been cited as a major barrier to use [16–18]. This study, however, found that the perceived heat and feelings of suffociation were associated with the insecticide and lack of airing out the net before use, not heat associated with a lack of moving air under the mosquito net. The literature states that these perceptions of heat from the insecticide may lead to lower net use, and some respondants agreed with that; however, other respondants requested additional education and communication messaging on the correct way air out their net so that the insecticide would dissipate and not overwhelm the user, potentially leading to net non-use.

### Limitations

There were multiple limitations in this study, as described in-depth elsewhere [21]. The nature of this qualitative study was meant to understand net use habits of a small number of the population. Due to this small sample, these results may not be representative of the community, region or country; they may not be generalizable to other contexts. Additionally, in Louga, and potentially in other regions of the country, respondents mistook the data collectors for those who distributed the nets during the UCC. Though the data collectors explained that they were independent from the UCC, there remains the possibility that respondents did not understand this distinction, potentially biasing responses. A limitation specific to this analysis includes confusion between the terms ITN and LLIN. While the intent was to ask about LLINs, when the questionnaire was translated into French and questions were further asked in Wolof, Peulh or Serere, the term *MILDA* or *moustiquaire* was generically used, referring to either an ITN or any type of net, respectively. However, since the UCC was ongoing during this research, it was assumed that most nets in Senegalese households were LLINs.

## Conclusion

Despite prior evidence that barriers such as heat, shape, insecticide and perceived mosquito density contribute to non-use of LLINs in other countries, this study has shown that these factors are considered more as nuisances and that they do not consistently prevent the use of nets among respondents in Senegal. Respondents appreciated and used their nets, adapting to the needs of their specific context. Key messages and clear communication around correct use and care and repair of nets, especially as it relates to insecticide, are needed. Additional research is also needed to better understand the role that behaviour change communication plays in the net culture of Senegal and how programme planners and policy makers can maintain and improve this culture. Finding ways to further minimize these barriers will help to ensure continued high use rates, and to further reduce malaria incidence in Senegal.
